# Parental Factors Associated with Rumination Related Metacognitive Beliefs in Adolescence

**DOI:** 10.3389/fpsyg.2017.00536

**Published:** 2017-04-10

**Authors:** Ka-wai Chow, Barbara C. Y. Lo

**Affiliations:** Department of Psychology, The University of Hong KongPokfulam, Hong Kong

**Keywords:** metacognitive beliefs, rumination, depression, adolescents, parenting

## Abstract

An increasing number of research studies have suggested that metacognition is associated with individuals’ mental health. Specifically, metacognitive beliefs about rumination was proposed to link to the onset and maintenance of depression according to the metacognitive model of depression. The current study aimed to serve as a pilot study exploring how parents’ metacognitive beliefs and parenting characteristics are associated with rumination related metacognitive beliefs in adolescents. Eighty-five parent–youth dyads were invited to complete a set of questionnaires examining their metacognitive beliefs about rumination followed by a difficult puzzle task, in which parent–adolescent interaction patterns were recorded to examine the parenting style. Results found that parents’ and adolescents’ positive metacognitive beliefs about rumination were significantly associated with each other. In addition, parental negativity was significantly associated with adolescents’ positive metacognitive beliefs of rumination and parental over-involvement was marginally associated with adolescents’ negative metacognitive beliefs of rumination. The findings highlighted the association between parental factors and adolescents’ metacognitive beliefs about rumination. Implications on the prevention of adolescent’s depression were discussed.

## Introduction

Depression is one of the most prevalent mental health conditions occurring during adolescence, with a 1-year prevalence rate of 4–5% (Costello et al., 2005, 2006; March et al., 2007), and affecting around one quarter of adolescents all over the world (Verstraeten et al., 2009). Merikangas et al. (2010) found that by the age of 17–18, 15.4% of adolescents have met the clinical criteria for major depressive disorder or dysthymia. Significant public health concerns have been raised, since adolescents’ depression is associated with a series of negative outcomes, including academic problems, physical health problems, substance abuse, tobacco use, high-risk sexual behavior and impaired social relationships (Lewinsohn et al., 1994; Birmaher et al., 1996; Brent et al., 1998; Le et al., 2003). Suicidal risk is enhanced by 30-fold during adolescence (Stolberg et al., 2002). Adolescents’ depression is also linked to several long-term mental health problems. Prospective studies have found that on recovery from a depressive episode, children and adolescents may still continue to display subclinical depressive symptoms, negative attributions, impairment in global functioning and interpersonal relationships, increased smoking and physical problems (Kandel and Davies, 1986; Nolen-Hoeksema et al., 1992; Puig-Antich et al., 1993; Strober et al., 1993; Rohde et al., 1994; Rao et al., 1995). These detrimental impacts have called for attention to identify risk factors of adolescents’ depression at an early stage and an urge for developing intervention initiatives.

Rumination has been extensively studied as it contributes a key role associating with the onset, progression and persistence of individual’s depressed mood (Nolen-Hoeksema, 2000; Broderick and Korteland, 2004). It is an emotion coping strategy characterized by the tendency to continuously dwell on the past, particularly the negative experience, feelings, and the possible causes and consequences of an experience (Nolen-Hoeksema, 1987, 1991). Research on adolescents has found a significant association between rumination and depressive symptoms (Hart and Thompson, 1996; Abela et al., 2004; Papadakis et al., 2006; Muris et al., 2009; Chen and Li, 2013). Several longitudinal studies have further confirmed that rumination predicts the development and severity of depressive symptoms over time among adolescents (Schwartz and Koenig, 1996; Abela et al., 2002; Broderick and Korteland, 2004; Burwell and Shirk, 2007; Nolen-Hoeksema et al., 2007; Abela and Hankin, 2008, 2011; Abela et al., 2009; Rood et al., 2009; Verstraeten et al., 2009; Hilt et al., 2010). In an attempt to expand the understanding of the etiology of depression from a metacognitive perspective, Wells (2002) has proposed the metacognitive model of depression to explain the psychopathological pathways associating with rumination and depression processes.

The metacognitive model of depression highlights the role of metacognition in initiating rumination, which subsequently becomes a causation factor in triggering and maintaining depression. Metacognition refers to individuals’ capability to recognize, to control and to monitor their own cognitive processing (Schneider, 2008). Developmental studies examining metacognitive abilities in youths suggested that the core aspects of metacognitive skills, such as monitoring of one’s own declarative memory knowledge as well as self-control of goal regulation strategies, begin to develop from early preschool years and mature steadily in late childhood and adolescence (see Schneider, 2008 for review). A recent research conducted by Weil et al. (2013) examined the developmental changes associated with metacognitive monitoring of self-performance in a cognitive task. The results also indicated that such reflective ability improves with age across the period of adolescence, in which it optimizes in late adolescence and plateaus in young adulthood. The authors argued that adolescence marks a key stage for investigating self-awareness of one’s own thoughts and behaviors, which in consequence draws important implications on development of many other aspects in life. This postulation echoes with the results of a research done by Palladino et al. (1997), who studied individual difference associated with adolescents’ cognitive styles and its relations with metacognitive ability and depression. The study found that adolescents (age 11–13 years old) who adopt an impulsive cognitive style tended to show poorer metacognitive monitoring skills and exhibit higher depressive level at the same time. The relationship of how metacognition may link to depressive outcomes, however, was not uncovered in this study. In an attempt to address this question, Wells and colleagues suggested that one option was to focus on metacognitive beliefs about rumination (Papageorgiou and Wells, 2001a,b; Wells, 2002).

In the metacognitive model of depression, metacognitive beliefs about rumination were hypothesized to be a key factor to activate a ruminative process and thereby inducing occurrence of depressive mood (Wells, 2002). Those beliefs are classified as positive and negative based on their contents (Papageorgiou and Wells, 2001a). Positive metacognitive beliefs refer to the advantages and usefulness of rumination (e.g., “Ruminating about my depression helps me to understand past mistakes and failures,” “I need to ruminate about my problems to find the causes of my depression”). On the other hand, negative metacognitive beliefs are related to the negative consequences arising from rumination. These negative consequences include negative emotional experiences and feelings of harm and uncontrollability (e.g., “When I ruminate I can’t do anything else”), and the individual’s interpretation of negative social consequences (e.g., “Everyone would desert me if they knew how much I ruminate about myself”). The metacognitive model of depression proposed that positive metacognitive beliefs about rumination would foster the adoption of ruminative strategies in response to stress and negative events. Once the cycle of sustained rumination begins, negative beliefs of rumination would then develop and intensify the negative mood, eventually leading to the onset of depressive symptoms (Papageorgiou and Wells, 2003).

The association between metacognitive beliefs about rumination, rumination and depression is supported by several cross-sectional and longitudinal studies (Papageorgiou and Wells, 2001b, 2003, 2009; Roelofs et al., 2007; Roelofs et al., 2010; Weber and Exner, 2013). Yet it appears that no previous research has systematically examined the developmental antecedent factors associated with the formation and origins of metacognitive beliefs about rumination. Notwithstanding the lack of research, theoretical assumptions on the formation of metacognitive beliefs can be deduced based on existing theories and researches conducted in akin area such as cognitive styles acquisition.

Cognitive style and metacognitive beliefs shared similar features in that they are all associated with a set of values or mental representations toward specific latent concepts. Specifically, cognitive style is a broad term depicting individuals’ preference to organizing and processing information (Riding and Pearson, 1994), including examples like cognitive schema and attributional style. Cognitive schema refers to a set of mental representations for the world or a system, which helps individuals perceive and organize new information (Piaget, 1952). Attributional style, on the other hand, describes an individual’s tendency to interpret or appraise the causes of a situation or circumstance in a particular way (Ball et al., 2008).

In the literature, learning theories have been proposed in an attempt to explain how formation of cognitive styles may arise through social interaction. Situated cognitive theory (Brown et al., 1989) for example suggested that individual’s knowledge and concept formation are framed and influenced by the activity, context, and culture in which the knowledge was adapted and utilized. It postulated that learning is not an isolated process, since people acquire and refine knowledge, beliefs, language, and problem solving skills through consistent shaping processes that naturally occur in social engagement, sharing and discussion. In line with this idea, social learning theory (Bandura, 1977) suggested that individual acquisition of knowledge happened within social contexts, by viewing learning as a dynamic cognitive process linking relationship or contingency between an observed behavior as well as the consequences of the behavior (i.e., vicarious reinforcement). In this framework, the observer is not merely a passive receiver during the learning process, and instead he or she is both influencing and being influenced by other personal factors and the social environment (i.e., reciprocal determinism).

From childhood to adolescence, parents spent a lot of time interacting with their children. In consequence, children may acquire concept and knowledge from them through direct teaching, modeling, observation and various means in these interactions. Specifically, past studies have extensively examined two ways of parental influence on offspring’s cognitive styles, namely the direct modeling of parents’ beliefs and the influence of parenting on children’s beliefs.

Evidence regarding direct modeling of parents’ beliefs has been mixed. Some studies have found a correlation between children’s and parents’ attributional style and cognitive schema (e.g., Seligman et al., 1984; Stark et al., 1996; Alloy et al., 1999, 2006; Garber and Flynn, 2001). A few other studies contrariwise have shown an association between children’s and mothers’ but not fathers’ negative cognitive style, including attributional style for negative events (Seligman et al., 1984; Alloy et al., 1999), and negative cognitive triad (i.e., individual view of self, world and future) (Stark et al., 1996). The findings that children follow only their mother’s but not father’s cognitive style are attributed to the fact that mothers are the primary caregivers in most families (Seligman et al., 1984; Stark et al., 1996). This implies that children are more likely to model themselves with their primary caregivers. While there is some evidence supporting the direct impact of parents’ cognitive style, a few studies have also found inconsistent results. These studies observed no association between children’s and their parents’ attributional style (Kaslow et al., 1988; Turk and Bry, 1992; Garber and Flynn, 2001), nor the dysfunctional attitudes and self-schema (Oliver and Berger, 1992).

A second pathway concerns parenting influence on children’s belief formation (Hankin et al., 2009). Parenting aggregates parental attitudes, practices and non-verbal expression, which affect the parent–child interaction across various circumstances (Darling and Steinberg, 1993). Developmental theories have suggested that the quality of both the parent–child bond (Bretherton, 1985) and parenting practices (Baumrind, 1971, 1978) might facilitate children’s development in different domains, including beliefs and values formation (Kohlberg and Kramer, 1969; Kohlberg, 1976; Darling and Steinberg, 1993).

The suggested link between parenting and cognitive style has been extensively examined in several correlational and prospective studies. Researchers have found an association between children’s negative cognitive style and negative parenting. For instance, parents with high levels of criticism, rejection, and control, as well as low levels of warmth and acceptance, are linked to children’s and adolescents’ negative cognitive schema (Beck, 1967), as well as negative and self-blaming attribution style (Jaenicke et al., 1987; Blatt and Homann, 1992; Goodman et al., 2011). In addition, a significant association has been found between over-controlling parents and increased sense of uncontrollability, as well as a lowered sense of personal competence in their children (Chorpita et al., 1996, 1998; Barlow, 2002). This group of children also tends to perceive the environment as more hostile and threatening (Hudson and Rapee, 2001).

Up to now, no study has examined the association between parenting and children’s metacognitive beliefs about rumination. Nevertheless, among the various parenting characteristics, parental negativity (or rejection) and over-control (or over-involvement) are found to be highly related to children’s and adolescents’ depressive symptoms (Burbach and Borduin, 1986; Marton and Maharaj, 1993; Kaslow et al., 1994; Chiariello and Orvaschel, 1995; Rapee, 1997; Garber and Flynn, 2001; McLeod et al., 2007). Parental negativity (or rejection) refers to a parenting style with a high level of criticism and disapproval, a low level of parental warmth and affection, as well as a lack of positive contact with the children (Rohner, 1986; Maccoby, 1992; Gerlsma and Hale, 1997; Rapee, 1997; Clark and Ladd, 2000). Past studies have suggested that highly rejecting parents would increase children’s cognitive vulnerability to depression. Children growing up under a highly critical environment tend to have a low self-esteem and a heightened sense of helplessness, which foster the development of a negative self-schema (Hammen, 1992; Kaslow et al., 1994; Garber and Flynn, 2001). In addition, parental negativity (or rejection) is also found to be associated with passive or emotion-focused coping, such as rumination, among children and adolescents (Hammen, 1992; Kaslow et al., 1994; Garber and Flynn, 2001).

Parental over-control (or over-involvement) is signified by an excessive involvement in children’s routines and emotional experiences, which encourage the children to rely on their parents (Barber, 1996; Ballash et al., 2006; McLeod et al., 2007). This form of parenting may limit the children’s access to the alternative solutions and deliver a message to the children that the world is unsafe, or that they are incapable to deal with the external threat. Children growing up under such parenting environment are found to possess a lower level of perceived mastery (Chorpita and Barlow, 1998) as well as poorer locus of control (Magaro and Weisz, 2006), and to develop a heightened sense of perceived helplessness (Kaslow et al., 1994; Garber and Flynn, 2001).

Given the close relationship found between parental negativity and parental over-involvement with the development of depression and rumination in children and adolescents in past literature, one would wonder if they may be impacting upon the development of metacognitive beliefs of rumination as well. The current study intended to serve as a pilot study to explore the relationship between parental factors (i.e., parent’s metacognitive beliefs and parenting styles) and metacognitive beliefs among adolescents in view of existing research gap. First, we would examine the association between parents’ and adolescents’ metacognitive beliefs about rumination. Given that previous literatures have suggested a strong association between parents’ and offspring’s cognitive styles, it was hypothesized that a significant correlation would be found between parent’s and adolescents’ metacognitive beliefs. Second, it is also hypothesized that parental negativity and involvement may significantly correlate with adolescents’ positive and negative metacognitive beliefs about rumination.

## Materials and Methods

### Participants

The sample consisted of 85 parent–adolescent dyads from local secondary schools. The participants were recruited by sending invitation letters to 40 randomly selected local secondary schools in Hong Kong. Of these, two schools agreed to participate in the study. Subsequently parental consent forms were distributed to all Grade 7 to Grade 9 students and their parents (specifically addressing to adolescents’ primary caregiver) in the two schools. A total of 158 parents and 85 adolescents consented to take part in the study. The mean age of the primary caregivers (63 female, 22 male) was 45.0, ranging from 30 to 61 with a standard deviation of 5.67-years old. The mean age of the adolescents (50 female, 35 male) was 13.2, ranging from 11 to 16 with a standard deviation of 1.2-years old. Fifty adolescents aged between 11 and 13 (i.e., in early adolescence); and 35 of them aged between 14 and 16 (i.e., in mid-adolescence).

### Measures

#### Questionnaires

##### Metacognitive beliefs about rumination

The Positive Beliefs about Rumination Scale (PBRS) (Papageorgiou and Wells, 2001b) and Negative Beliefs about Rumination Scale (NBRS) (Papageorgiou and Wells, 2001a) were administered to assess participants’ metacognitive beliefs about rumination. They were asked to rate the extent to which they agreed with each item based on a four-point Likert Scale ranging from 1 (do not agree) to 4 (agree very much). The PBRS consists of nine items measuring positive metacognitive beliefs regarding the benefits and advantages of rumination (e.g., “I need to ruminate about my problems to find answers to my problems”). Total scores fall between 9 and 36. The original PBRS has demonstrated good internal consistency, test–retest reliability, and convergent and concurrent validity (Papageorgiou and Wells, 2001a,b). The NBRS consists of 13 items measuring negative metacognitive beliefs about rumination. The negative metacognitive beliefs are related to the sense of uncontrollability (e.g., “Rumination about my problem is uncontrollable”) and harm (e.g., “Rumination makes me physically ill”), as well as the negative interpersonal consequences that are associated with rumination (e.g., “People will reject me if I ruminate”). Total scores fall between 13 and 52. The original NBRS demonstrated good internal consistency, test–retest reliability, and convergent and concurrent validity (Papageorgiou and Wells, 2001a; Luminet, 2004).

Both the original PBRS and NBRS were translated into Chinese in the present study. The translation method was adopted from Ng and Bhugra (2008), which had also applied PBRS and NBRS in a Chinese population. The items were first translated into Chinese by the first author and backward translated into English by a bilingual PhD in clinical psychology student not involved in the current study. Three Ph.D. in psychology students were invited to rate the level of agreement between the original and back translated versions. Kappa agreement between the three students for PBRS was 91% and for NBRS was 89%. The Chinese version of the PBRS and NBRS also demonstrated good reliability in the current sample. For PBRS, Cronbach’s alpha of 0.88 was obtained for the adolescent sample; and 0.93 for the adult sample. For NBRS, Cronbach’s alpha of 0.83 was obtained for the adolescent sample, and 0.89 for the adult sample.

### Procedures

Consented parents and adolescents were contacted via phone to schedule a time for testing at a psychology laboratory. Upon arrival, they were led to the interviewing room by the investigator and were briefed about the procedures of the experiment. The experiment consisted of two parts, including completion of questionnaires in part 1 and an observation task in part 2.

In the first part, parents and adolescents were given PBRS and NBRS to fill in, which took approximately 20 min to complete. They were asked to fill in the questionnaires anonymously. Each of them was assigned a code for identification and matching purposes. Demographic information including age, sex and date of birth was collected. They were asked to choose one answer per question and double check to avoid any missing questions.

After completing the questionnaires, adolescents were invited to perform a tangram task based on the protocol developed by Hudson and Rapee (2001, 2002). The tangram task has been conducted in an Asian sample in a cross-cultural study (Oh et al., 2002). The parent (i.e., primary caregiver) was asked to stay with their child during the task for support. Adolescents were given a set of tangram, that is, a dissection puzzle consisting of seven geometrical pieces. They were then shown a paper printed with the silhouette of five shapes. Adolescents were instructed to use all the seven pieces to form each specific shape one by one, and were asked to complete the task in 5 min. The parent was presented with the following instruction before the commencement of the task.

The test is to examine your child’s cognitive performance; he/she has 5 min to complete five diagrams. He/she needs to complete each diagram with all the seven pieces. Most students at their age would find it easy, but some may find it difficult. I will give you the answer sheet. You can assist your child if you think he/she really needs help. If you do not have any questions, your child will begin the task when I press the timer.

A timer was placed on the desk so that the parent–adolescent dyad could monitor the time. A camera was placed in front of them and the entire 5-min interaction was videotaped. When the time was up, the investigator debriefed the participants about the objective of the task. It was fully explained that the tangram task was designed to observe their interaction and the problems were intentionally designed to be difficult such that most adolescents might not be able to complete it within the given time. After the debriefing, each parent–adolescent dyad was given HKD$100 (approximately US$13) in appreciation of their time spent.

#### Observations

The 5-min parent–adolescent interaction was coded based on the protocol developed by Hudson and Rapee (2001, 2002). The interaction was rated on nine global scales measuring the degree of parental involvement and negativity. The global scale involved a nine-point continuum ranging from zero to eight, with four representing the neutral point. The nine scales were developed to measure two latent factors: Involvement and Negativity. The Involvement factor was calculated by averaging the mean of five scales, including (1) degree of unsolicited help (e.g., whether parents offer no help to the adolescent at all or offer excessive and unnecessary help); (2) general degree of parental involvement/help (e.g., the degree to which parents participate in the task); (3) touching of the tangram pieces (e.g., how frequent parents physically touches and move the tangram pieces); (4) parent’s posture (e.g., whether parents sit right back or lean on the table during the task); (5) parent’s focus during the interaction (e.g., whether parents attend to the adolescent or the task more). A high score of Involvement factor represented parents being overly involved and intervening with the task in an intrusive manner. A low score of Involvement factor indicated that parents were involved in the task with a less intrusive manner, and adolescents were given more opportunity and autonomy to attempt the task on their own.

The Negativity factor was calculated by averaging the mean of four scales, including (6) degree of parent’s positive affect (e.g., whether parents’ facial expression, body language appear to be happy or angry during the task); (7) general mood/atmosphere of the interaction (e.g., whether the overall interaction appears to be comfortable or tense); (8) parent’s tension (e.g., whether parents’ behavior and gestures appear to be relaxed or tense); (9) degree of parent’s verbal/non-verbal encouragement/criticism (e.g., how frequent parents give encouragement or negative comments to the adolescent). High Negativity scores indicated parents being more negative and critical toward the adolescent. Low Negativity scores represented parents being more positive and encouraging during the parent–adolescent interaction. In general, a lower rating on the scales represented more positive parent–adolescent interactions (i.e., a lower level of Involvement and Negativity), while a higher rating on the scales represented a more negative parent–adolescent interaction (i.e., a higher level of Involvement and Negativity).

Two students, one undergraduate student and another postgraduate student viewed and rated the videos. Both of them studied psychology and were blinded to the hypotheses of this study. They were trained for 10 h before conducting the coding. The undergraduate coder completed the coding of all videos, while the postgraduate coder rated 50% of the video to ensure that the ratings were made in alignment with the protocol guideline. Kappa was used to calculate the categorical agreement between the two raters. The inter-rater reliability of the Involvement factor obtained was 0.82, and that of the Negativity factor was 0.80.

## Results

### Correlation Analysis

Mean and standard deviations of the variables are presented in **Table 1**. Positive association was found between parents’ and adolescents’ positive metacognitive beliefs (*r* = 0.33, *p* < 0.01) as well as between parent’s and adolescents’ negative metacognitive beliefs (*r* = 0.23, *p* = 0.03) (see **Table 2**). In considering Bonferroni corrections of the alpha level when comparing four correlations (i.e., *p* = 0.0125), only significant positive correlation was found between parents’ and adolescents’ positive metacognitive beliefs.

**Table 1:** Descriptive statistics of parents’ and adolescents’ PBRS, NBRS, parental involvement, and parental negativity.

	Mean	Standard deviation
Adolescents’ PBRS	21.66	5.73
Adolescents’ NBRS	18.15	5.14
Parents’ PBRS	21.36	6.48
Parents’ NBRS	19.67	6.49
Parental involvement	4.41	1.82
Parental negativity	3.31	1.48


*PBRS, Positive Beliefs about Rumination Scale; NBRS, Negative Beliefs about Rumination Scale.*

**Table 2:** Correlation between parents’ and adolescents’ PBRS, NBRS, parental involvement, and parental negativity.

	Adolescents’ PBRS	Adolescents’ NBRS	Parents’ PBRS	Parents’ NBRS	Parental involvement	Parental negativity
Adolescents’ PBRS	–	0.02	0.33^∗∗^	0.01	0.14	0.28^∗∗^
Adolescents’ NBRS	–	–	-0.08	0.23^∗^	0.26^∗^	0.14
Adolescents’ PBRS	–	–	–	-0.00	0.04	-0.07
Adolescents’ NBRS	–	–	–	–	0.03	0.20
Parental involvement	–	–	–	–	–	0.03
Parental negativity	–	–	–	–	–	–


*PBRS, Positive Beliefs about Rumination Scale; NBRS, Negative Beliefs about Rumination Scale. ^∗^*p* < 0.05; ^∗∗^*p* < 0.01 (two-tailed).*

**Table 2** presents the association between parenting characteristics and adolescents’ metacognitive beliefs. A significant positive correlation was found between adolescents’ positive metacognitive beliefs and parental negativity (*r* = 0.28, *p* < 0.01), but not between adolescents’ positive metacognitive beliefs and parental involvement (*r* = 0.14, *p* > 0.05). The correlation between adolescents’ negative metacognitive beliefs and parental involvement was marginally significant (*r* = 0.26, *p* = 0.02) when Bonferroni corrections of the alpha level were considered. On the other hand, the correlation between adolescents’ negative metacognitive beliefs and parental negativity was not significant (*r* = 0.14, *p* > 0.05).

## Discussion

The present study was one of the first to study the relationship between parental factors and metacognitive beliefs about rumination in adolescents. Our results suggested that parents’ and adolescents’ positive metacognitive beliefs were found to significantly correlate with each other. Moreover, parental negativity was found to significantly correlate with adolescents’ positive metacognitive beliefs whereas parental over-involvement was marginally correlated with adolescents’ negative metacognitive beliefs.

Our findings first of all provide preliminary support that adolescents might be able to acquire rumination related metacognitive beliefs directly from parents. The present results are in line with the social learning theory and previous studies finding significant association between children’s and parents’ cognitive styles (Seligman et al., 1984; Stark et al., 1996; Alloy et al., 1999; Garber and Flynn, 2001). Social learning theory (Bandura, 1977) suggested that knowledge acquisition happened within social contexts. Since a child spend most of the time interacting with his/her parents since young, it is reasonable to believe that parents, particularly the primary caregiver, are significant social agents in facilitating the child’s belief formation through various means. Previous study has proposed that children could model parent’s beliefs through verbal means of communication (Schwartz, 2006). For example, it was found that parental instruction could foster children to develop knowledge about task-specific strategies (i.e., metacognitive beliefs about task-specific strategies). Specifically, Carr et al. (1989) and Kurtz et al. (1990) found that children tend to display increased use of strategies (e.g., rehearsal, categorization) to complete sort-recall tasks when their parents give appropriate instructions about such strategies, and the children also showed a better memory of these instructed strategies at a later time. This reflects that the parental instructions may shape the children’s understanding of the strategies’ application. In other words, the parents’ verbal instructions facilitated the internalization of metacognitive beliefs about these strategies. In a similar vein, parents could possibly transmit their positive metacognitive beliefs about rumination to their children through a verbal mean. For instance, by telling their child “It is necessary to reflect deeply about the reason for failing the test because it will help you improve.” When parents repeatedly express their metacognitive beliefs to their children, children might eventually internalize these beliefs. Future study could adopt an observational approach to study the modeling of metacognitive beliefs about rumination, as well as the proposed underlying mechanism(s) involved, in this respect.

Interestingly, the present findings indicated that the association between parents’ and adolescents’ metacognitive beliefs of rumination was significant in the case of positive metacognitive beliefs but not in negative metacognitive beliefs when Bonferroni corrections were considered. According to Papageorgiou and Wells (2001a,b, 2003), positive metacognitive beliefs mainly concern the usefulness or advantages of rumination as a coping strategy (e.g., “Rumination helps me understand my past mistakes and failures”). Parents with their views and beliefs on this may transcend to their children through pathways described above. Negative metacognitive beliefs of rumination on the other hand concerns mainly about the negative consequences arising from rumination (e.g., “Everyone would desert me if they knew how much I ruminate about myself”). The items tap into the experiential knowledge (particularly the negative aspects) when one has adopted rumination as a preferential coping strategy. In the present study, our recruited community adolescent sample had a relatively low score on NBRS, with a mean of 18 out of the potential range of 13–52. The low endorsement rate and limited variance may have impacted on strength of association between parents’ and adolescents’ negative metacognitive beliefs. Future study should include rumination and depression scales as covariate controls, or to recruit a clinically depressed comparison sample to examine if the pattern of results may differ across other populations.

A second major finding of the study relates to the observation that parenting characteristics may be associated with adolescents’ metacognitive beliefs. In particular, a high level of parental negativity was associated with a high level of positive metacognitive beliefs about rumination in adolescents. Parents who scored high on parental negativity during the tangram task displayed more criticism, rejection and discouragement to their children. When being nourished under such negative and critical environment, adolescents may be likely to internalize their emotion and become less motivated to adopt active (or problem solving) coping strategies (Hammen, 1992; Kaslow et al., 1994; Garber and Flynn, 2001), because further expression of emotion and active coping (e.g., help seeking behavior) may result in more parental rejection and negative responses (Eisenberg et al., 1998). Adolescents thereby appear likely to develop positive metacognitive beliefs about the usefulness of passive (or emotion-focused) coping strategies, such as rumination, believing that focusing more on their emotional experience would be helpful to regulate their emotion; or at least that these copings would be functional and not to trigger or initiate further negative reactions from parents. These beliefs would then further encourage the use of more passive coping strategies. The development of positive metacognitive beliefs and increased use of passive coping eventually may enhance their risk to depression according to the metacognitive model of depression (Wells, 2002).

The present study also observed that the highly involved parents often offered help repetitively to their children during the tangram task, despite having no obvious sign that their children might need help. In some instances, the participating adolescents even rejected the help from parents because they wanted to attempt the task on their own first. The over-involved parents in consequence might limit the opportunity for their children to explore the external environment. The lack of opportunity to explore the outside world is suggested from previous studies to foster the development of a sense of incompetence, if it happens consistently (Chorpita et al., 1996, 1998; Hudson and Rapee, 2001; Barlow, 2002). Adolescent are likely to possess lower levels of perceived mastery (Chorpita and Barlow, 1998) and locus of control (Magaro and Weisz, 2006), as well as to exhibit a heightened sense of perceived helplessness (Kaslow et al., 1994; Garber and Flynn, 2001) when parents intervene their daily activities in an intrusive manner. This may in turn promote a sense of uncontrollability toward their cognitive process and emotional experience. Hence, they may be more prone to develop the negative metacognitive beliefs about rumination, which in turn may intensify and prolong the depressive mood according to Wells (2002) model. In the present study, only a weak relationship was observed between parental over-involvement and adolescents’ negative metacognitive beliefs of rumination. More future research evidence again would be needed to examine if the results are replicable and can be generalized to clinically depressed adolescent populations.

Following from the previous point, it is noteworthy that the correlation sizes observed in the present study were rather small, ranging from 0.23 to 0.33 (**Table 2**), which implied that there may be other factors associated with the metacognitive beliefs development in adolescents aside from the parental factors observed. Specifically, adolescence concerns a stage of self-identity formation (Erikson, 1950), with increased exploration in the social environment and outside world. Peer influence and relationships in other social domains appear to be influential in addition to the familial factors. For example, previous studies have suggested that peer influence was one of the significant factors affecting adolescent’s cognitive development and behaviors. Meeus and Dekovic (1995) found that peers are more influential on the development of adolescent’s identity as compared to their parents. Hence, future research could consider extending the present findings and explore how peers or other possible socialization factors may be associated with development of adolescents’ metacognitive beliefs.

The clinical implications of the present findings imply that parents could be considered as candidates for therapeutic targets and the beneficial effects might be two-fold. First, it might be beneficial to implement metacognitive training on parents as a preventive measure against both adult’s and adolescent’s depression. When parents are able to equip themselves with more adaptive metacognitive beliefs about rumination, it could reduce their vulnerability to depression, and also reduce the chance for the next generation acquiring those beliefs from them. Past studies have demonstrated that depressed adults were responsive toward the metacognitive therapy (Nordahl, 2009; Wells et al., 2009, 2012; Ashouri et al., 2013; Jordan et al., 2014; Dammen et al., 2015; Mami et al., 2015; Papageorgiou and Wells, 2015), in which patients showed decreased level in metacognitive beliefs about rumination after the therapy. Therefore, it appears that parents’ metacognitive beliefs could be modified when appropriate trainings are provided.

Second, the metacognitive training for parents could include parenting skills, which may also facilitate more adaptive parenting behaviors (i.e., avoiding negative and over-controlling behaviors). Avoiding those maladaptive parenting responses is likely to benefit children’s mental health in several ways. These include reducing the chance that the children may develop negative cognition, adopt passive coping strategies (Muris et al., 2001; Spasojević and Alloy, 2002; Meesters and Muris, 2004; Brumariu and Kerns, 2010), and develop metacognitive beliefs about rumination, thereby reducing their risk to developing depression.

## Limitations and Future Directions

There were several limitations in the current study. First, the sample size of the current study was relatively small with unequal age composition and gender distribution. The current sample consists of more participants in early adolescence than in mid-adolescence, as well as more girls than boys. Several studies suggested that rapid development of the brain (i.e., substantial changes in the cognitive, affective and behavioral systems) happens in early adolescence (Sowell et al., 2002; Gardner and Steinberg, 2005; Paus, 2005; Blakemore and Choudhury, 2006; Steinberg, 2008), and that boys and girls tend to undergo different neurological change during this stage (Blakemore and Choudhury, 2006). Due to the small sample size, the current study was unable to further divide the sample into sub-groups across age and gender. Future study may consider recruiting a larger community sample in balancing the age and gender distributions to test these effects.

Moreover, the current study did not include any measure of rumination and depression of parents and adolescents. Future study could introduce such measures to control for these effects. Likewise, there was also a limitation in our study design regarding the use of single observation method to measure parenting. Although the parenting behavior observation method adopted in the present study had been previously validated in another Asian sample (Oh et al., 2002), it would be appropriate to administer additional parenting questionnaire to cross-check the rated observations to avoid mono-method-biases. Furthermore, it is understandable that the parents might be more vigilant or inhibited under video recording procedure (i.e., reactivity bias), which may not accurately reflect the parenting behaviors displayed in daily context. Future study may consider examining the parent–adolescent interaction across various settings and situations, including home observation or other naturalistic settings to test the consistency of parenting styles.

Finally, an important point to note is that the current study was cross-sectional in nature, so no causal relationship could be inferred. That is, the current study only suggested that parental factors, including parents’ own metacognitive beliefs and parental characteristics, are associated with adolescent’s metacognitive beliefs. However, we could not confirm whether these parental factors would influence the metacognitive beliefs formation in adolescents. Future study could investigate the predictive relationship, as well as the potential mediating mechanism(s) involved, using a prospective longitudinal design.

## Conclusion

In summary, the present study demonstrated significant association between parental factors and adolescents’ metacognitive beliefs. Adolescents’ and parents’ positive metacognitive beliefs are found to significantly relate to each other. Parenting characteristics of negativity would significantly associate with adolescents’ positive metacognitive beliefs about rumination, and parental over-involvement was marginally associated with adolescents’ negative metacognitive beliefs about rumination. The present study expands the understanding of the metacognitive model of depression by examining the factors associated with metacognitive beliefs of rumination in adolescents. The findings provide preliminary foundation for follow-up work in designing novel and effective prevention program against adolescents’ depression from metacognitive and developmental perspectives.

## Ethics Statement

This study was carried out in accordance with the recommendations of Human Research Ethics Committee, University of Hong Kong with written informed consent from all subjects. All subjects gave written informed consent in accordance with the Declaration of Helsinki. The protocol was approved by the Human Research Ethics Committee, University of Hong Kong.

## Author Contributions

KWC contributed to study design, data collection and processing, statistical analysis, interpretation and paper writing. BL contributed to study design, statistical analysis and consultation, supervision, paper writing and review.

## Conflict of Interest Statement

The authors declare that the research was conducted in the absence of any commercial or financial relationships that could be construed as a potential conflict of interest.
